# Long-term culture of human pancreatic islets reveals reduced metal ion pathways in their gene signature

**DOI:** 10.1177/09636897251390960

**Published:** 2025-11-22

**Authors:** Hiroyuki Kato, Tara K. Sigdel, Mona Sheta, Keiko Omori, Meirigeng Qi, Fouad Kandeel, Hirotake Komatsu

**Affiliations:** 1 Transplant Surgery, Department of Surgery, University of California, San Francisco, San Francisco, CA, USA; 2 Department of Translational Research & Cellular Therapeutics, Arthur Riggs Diabetes & Metabolism Research Institute of City of Hope, Duarte, CA, USA

**Keywords:** pancreatic islets, islet transplantation, long-term culture, metal ion homeostasis, metallothionein, gene expression profiling

## Abstract

Pancreatic islet transplantation is an effective therapy for type 1 diabetes; however, its broader clinical application is limited by the shortage of donors. Establishing long-term culture methods for isolated islets is an area of ongoing investigation that may ultimately support applications such as biobanking and stem cell–derived islets. However, maintaining transplantable quality of islets during extended culture remains a challenge. We recently developed a method for human islet culture on optimally sized microwells that preserves viability over two weeks. Despite improved viability, other key pre-transplantation factors, such as islet metabolism, remained reduced, indicating a need for further optimization. To identify potential targets for improvement, we performed RNA sequencing on human islets from three deceased donors, comparing two-week cultures (microwell and conventional) versus pre-culture controls. Transcriptomic analysis showed significant gene expression changes in two-week-cultured islets compared to pre-culture islets, whereas microwell and conventional culture conditions showed minimal differences despite improved viability in microwell culture. Pathway analysis revealed that long-term culture consistently downregulates heavy metal ion–related pathways, particularly zinc-related pathways regulated by metallothioneins. This suggests a loss of β-cell characteristics during extended culture. Our findings highlight intra-islet metal ion homeostasis as a potential therapeutic target for improving transplantation outcomes following prolonged islet culture.

## Graphical abstract

**Graphical abstract:**
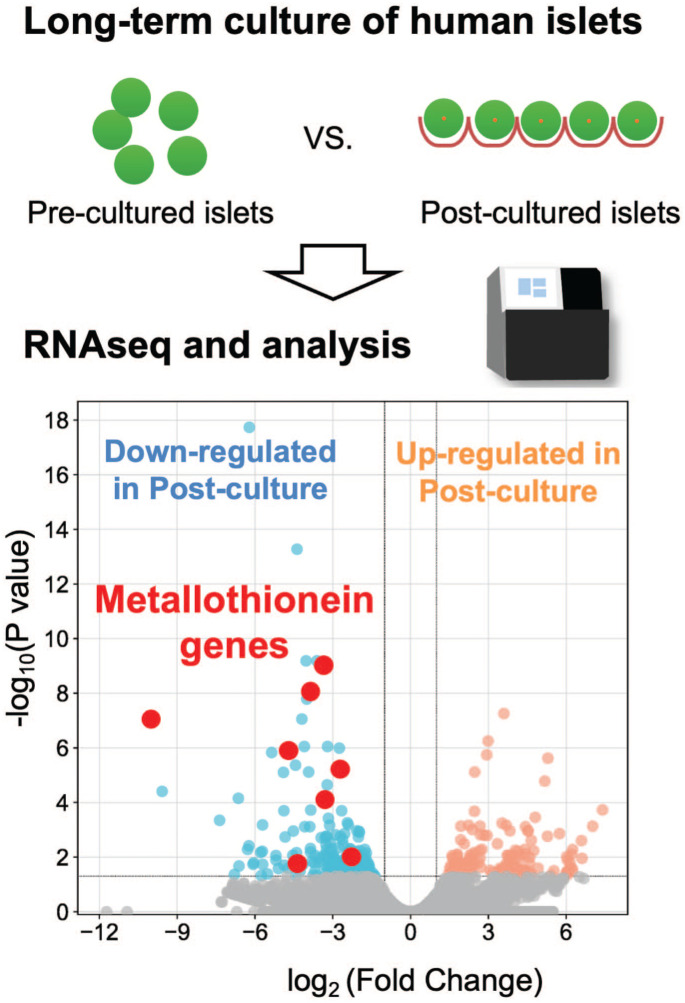

## Introduction

Beta cell replacement therapy is a promising treatment for type 1 diabetes, aiming to achieve improved glycemic control for patients^1–4^. Current clinical procedures rely on isolated pancreatic islets from cadaveric donors for transplantation into patients. These isolated islets are cultured prior to transplantation but should ideally be transplanted within a few days. This is due to the difficulty in maintaining both the quality and quantity of pancreatic islets outside their native pancreatic environment in conventional culture settings^5–7^. The establishment of robust long-term culture methods could provide additional advantages for the field. Such approaches may enable extended quality control and functional testing of islets before transplantation, to ensure selection of high-performing grafts. In addition, while donor organ availability remains the principal limitation of current islet transplantation, long-term culture could still provide indirect benefits. Robust culture systems would enable the establishment of islet banks, similar to stem cell or organoid repositories, facilitate pooled or staged transplantation, reduce the urgency associated with fresh organ use, and potentially improve equity of islet access.

For the successful clinical islet transplantation for type 1 diabetes patients, the number of transplanted islets is a major determinant of clinical outcomes, including the achievement of insulin independence^2,3,8^, clinical islet product release criteria after the isolation often include a minimum islet mass of more than 5,000 islet equivalents (IEQ) per kilogram of the recipient body weight^1^. Published reports and registry data further emphasize that while relatively low purity thresholds of ~30% to 50% are generally accepted for product release, achieving sufficient islet mass is operationally prioritized^9–12^. Therefore, a low yield of islets from a single donor is sometimes a limiting factor that prevents clinical transplant centers from proceeding with transplantation. While the clinical trend is to minimize the number of donors per recipient in order to reduce alloimmunization risk, future advances in immune modulation—such as the development of hypoimmunogenic or gene-edited islets—may broaden the safe use of pooled donor material. In this context, long-term culture could provide a practical means to preserve and combine islets from multiple isolations when single-donor yields are insufficient.

Long-term culture of isolated islets has been explored using various methods, including hydrogel-based systems, low temperature environments, and oxygenated conditions^7,13^. However, no long-term islet culture strategy has yet been realized in clinical practice. It is well recognized from experimental studies that islet viability significantly decreases over time, especially when islets fuse together, which limits the diffusion of nutrients and oxygen^5,14^. Since high islet viability in the pre-transplant quality assessment is one of the prediction factors for successful engraftment^15^, maintaining high viability remains the fundamental objective in developing long-term islet culture systems. To address this, we recently developed a simplified approach to mitigate the fusion of multiple islets during culture by physically separating individual islets using microwell plates^5^; we successfully achieved a two-week culture of primary isolated human islets with minimal necrosis and maintained islet mass, outperforming conventional culture methods with flat bottomed plates and even comparable to pre-culture conditions. Importantly, the microwell platform maintained insulin secretory function and the proportion of major endocrine cell types after long-term culture, suggesting that this approach preserves both viability and islet identity.

Despite improved viability, we identified one potential caveat in the long-term cultured islets: islet metabolism measured by oxygen consumption rate was reduced in microwell-cultured islets compared to pre-culture levels, with no improvement over conventional culture. In fact, islet oxygen consumption rate is another key functional metric—alongside viability—in pre-transplant assessments used to predict successful transplantation outcomes^16,17^. This suggests that further modifications to the microwell platform may be necessary to maintain islet metabolism and ultimately improve transplantation outcomes when using long-term cultured islets. To explore potential therapeutic targets, in this study, we analyzed gene expression profiles of long-term cultured islets—both microwell and conventional flat-dish cultures—as well as pre-culture islets, using whole transcriptome analysis via RNA sequencing (RNA-seq).

## Materials and methods

### Human islet preparation

All human islet isolations were conducted at the Southern California Islet Cell Resource Center at City of Hope^18,19^, and obtained through the Integrated Islet Distribution Program. Human pancreata from deceased donors were obtained with informed consent from the donor families or legal representatives and with approval from the Institutional Review Board of City of Hope (IRB #01046). Three islet batches were included in this study. Standardized characteristics, consistent with the recommendations of *Diabetes* and *Diabetologia*^20,21^, are summarized in Supplementary Table S1, including basic donor information (donor age, donor body mass index, donor HbA1c level, cause of death, and cold ischemia time [time between the cross-clamping for pancreas procurement to pancreas digestion for islet isolation]), islet score and grade^22^, and post-isolation islet number.

### Long-term culture of human islets

The two week-cultures of isolated islets were performed either on flat-bottomed, conventional dishes in a 35-mm dish format, or on microwell dishes in a 35-mm dish format (EZSPHERE, AGC Techno Glass, Yoshida, Japan) as previously published^5^. Briefly, 1000 IEQ / dish were cultured in a CO_2_ incubator at 27°C, with 1 mL per dish of CMRL 1066 culture media (Corning Life Sciences, Tewksbury, MA, USA) supplemented with 0.5% human serum albumin, 0.1 μg/mL insulin-like growth factor-1 (Cell Sciences, Newburyport MA, USA), 10 U/mL heparin sodium (Sagent Pharmaceuticals, Schaumburg, IL, USA), and Penicillin-Streptomycin-Glutamine (Gibco, Waltham, MA, USA). Culture media was replaced every three days.

### RNA-seq and subsequent data analyses

RNA was isolated from 200 IEQ of islets after the two week-culture (TRI Reagent, Molecular Research Center, Cincinnati, OH, USA, and Direct-zol RNA Microprep, Zymo Research, Irvine, CA, USA). Messenger RNA was isolated from total RNA using poly-T oligo-attached magnetic beads. The RNA was then fragmented, and first-strand cDNA was synthesized with random hexamer primers, followed by the second strand cDNA synthesis using either dUTP for directional library or dTTP for non-directional libraries. The library was assessed by Qubit and real-time PCR for quantification and bioanalyzer for size distribution detection. Quantified libraries were pooled and sequenced on Illumina platforms, according to effective library concentration and data amount. The clustering of the index-coded samples was performed according to the manufacturer’s instructions. After cluster generation, the library preparations were sequenced on Novaseq X Plus-25B and paired-end reads were generated.

Raw data (raw reads) of fastq format were firstly processed through fastp software. In this step, clean data (clean reads) were obtained by removing reads containing adapter, reads containing ploy-N and low-quality reads from raw data.

The reference genome and gene model annotation files were obtained directly from the genome database website using Homo Sapiens Grch38/hg38 as the reference genome. The index of the reference genome was built using Hisat2 v2.0.5 and paired-end clean reads were aligned to the reference genome using Hisat2 v2.0.5. We selected Hisat2 as the mapping tool for Hisat2 can generate a database of splice junctions based on the gene model annotation file and thus a better mapping result than other non-splice mapping tools. featureCounts v1.5.0-p3 was used to count the reads numbers mapped to each gene. Fragments Per Kilobase of transcript per Million mapped reads of each gene were calculated based on the length of the gene and reads count mapped to this gene. Only protein-coding genes were included, and genes with low variability (standard deviation < 0.25) were filtered out. The significance of differential expression was determined based on the log2 fold change and adjusted *p*-values (<0.05) calculated using the Benjamini-Hochberg method to control the false discovery rate.

Volcano plots were generated to visualize differentially expressed genes. The log2 fold change was plotted on the x-axis, and the -log10 transformed adjusted *p*-value was plotted on the y-axis.

Pathway enrichment analysis was performed using Enrichr (https://maayanlab.cloud/Enrichr/) for the biological processes, pathways and GO terms (adjusted *p*-value < 0.05).^23^

### Statistical analysis for the data of RNA-seq

Differentially expressed genes identification: Genes with |log2 fold change| > 2 and adjusted *p*-value ≤ 0.05 were considered significantly differentially expressed. For the gene expression changes in between Post-culture_Conventional and Post-culture_Microwell the *p*-value was calculated using unpaired *t*-test. For gene enrichment analysis, the false discovery rate <0.05 was considered as significant.

## Results

### Overview of the study design

**Figure 1.**
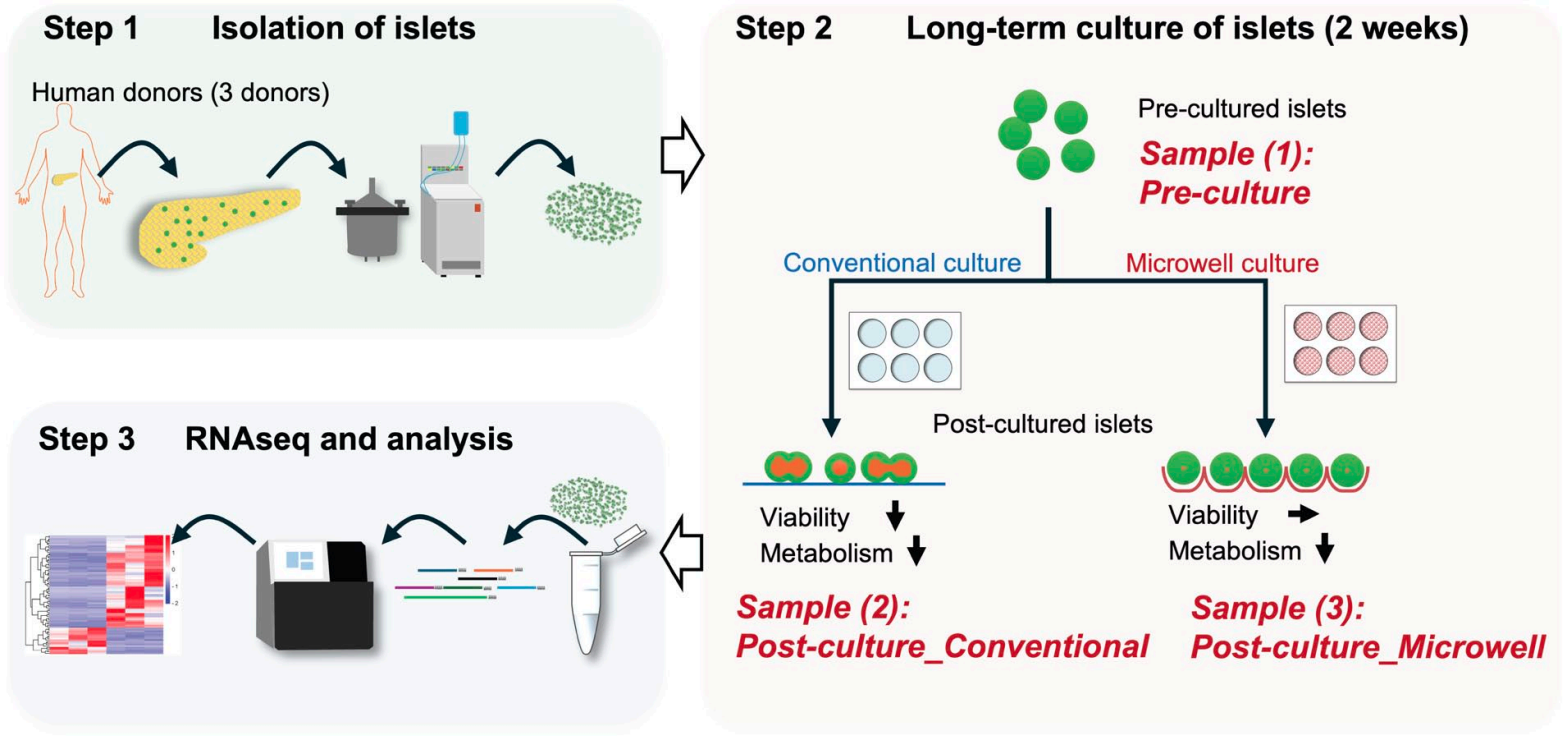
Overview of the study. Pancreatic islets were isolated from deceased donors, and long-term culture was performed using two different platforms: conventional culture plates and microwell culture plates. RNA samples were collected from (1) pre-cultured islets (Pre-culture), (2) post-cultured islets under conventional conditions (Post-culture_Conventional), and (3) post-cultured islets under microwell conditions (Post-culture_Microwell), for subsequent RNA sequencing analysis. The study included samples from three donors. This figure was created by the authors using Microsoft PowerPoint.

Fig. 1 presents an overview of the study. We prepared human isolated islets from deceased donors for the long-term culture (see details in the Materials and methods section and Supplementary Table S1). We collected human islet samples at two time points: before the long-term culture (Pre-culture) and after two weeks of culture. For the two-week culture, we employed two conditions: conventional flat-dish culture and microwell culture. We recently demonstrated that microwell culture improved the viability of long-term cultured islets compared to conventional culture^5^. In the study, while performing long-term culture of primary human islets with a focus on viability and function, we also collected islet samples for RNA extraction. The RNA obtained from these preparations was analyzed independently in the current study, separate from our previous publication. We collected RNA samples from the Pre-culture, Post-culture_Conventional, and Post-culture_Microwell groups for transcriptomic analysis using RNA-seq and pathway analysis.

### Long-term culture dominantly alters the gene expression profiles of isolated islets, while the culture method contributes to minimal changes

**Figure 2.**
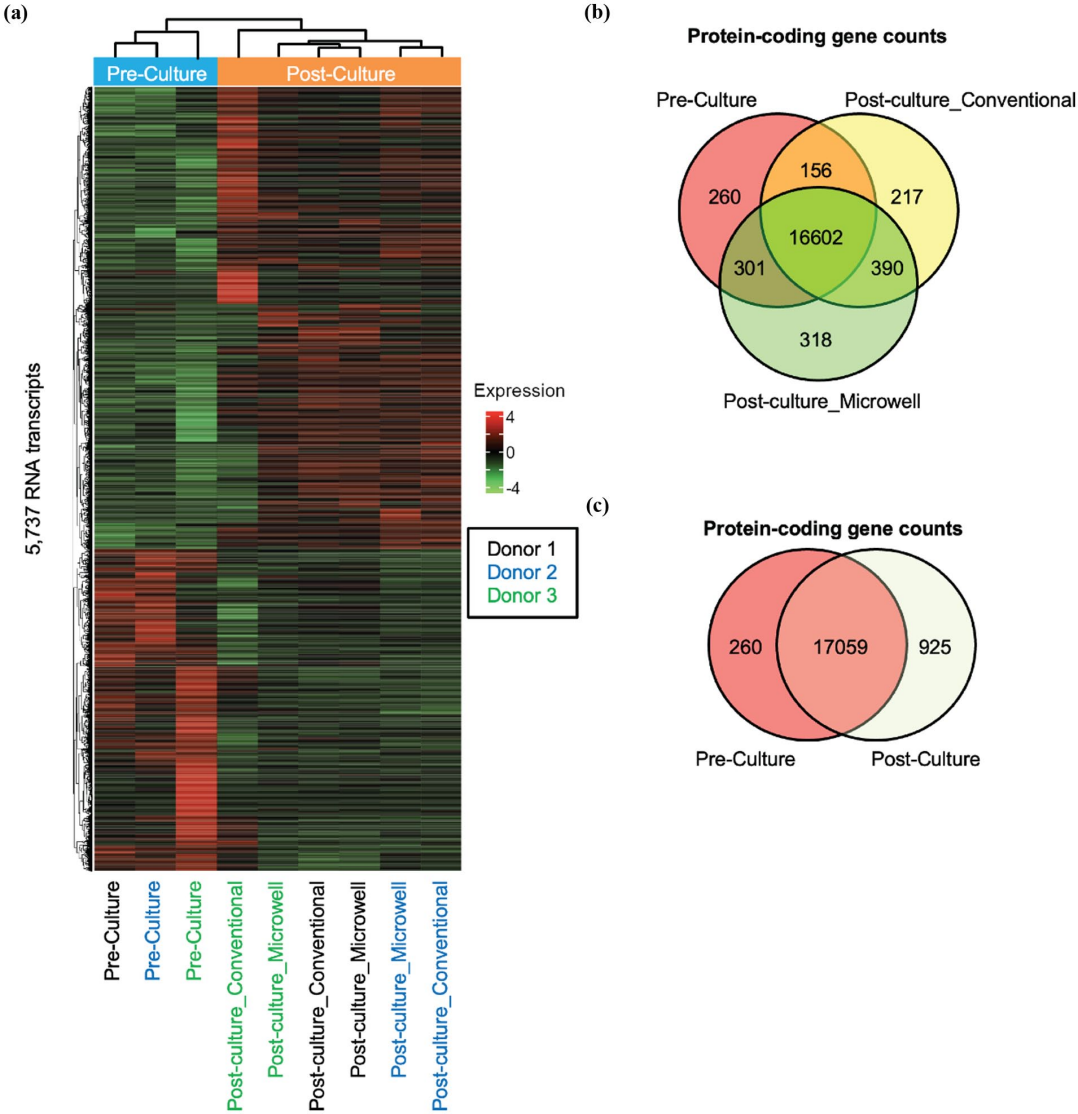
Overall gene expression signature of pre- and post-cultured islets. (a) Heatmap demonstrating the gene expression signature of nine individual samples from three human donors, along with clustering analysis. Donor IDs are indicated by distinct colored letters at the bottom of the heatmap. (b) Total protein-coding gene counts across the three groups: Pre-culture, Post-culture_Conventional, and Post-culture_Microwell. (c) Total protein-coding gene counts between the three groups: Pre-culture and Post-culture.

Across all nine samples analyzed, a total of 39,670 gene transcripts were sequenced. Of these, 18,244 were protein coding genes, and 651 were miRNAs. Thirty-seven RNA of mitochondrial origin were also sequenced (Supplementary Table S2). Analyzing all sequenced and captured transcripts including protein-coding, non-coding, miRNA, pseudogene and non-coding RNA for the perturbation, 5,737 RNAs were significantly different. Fig. 2a demonstrates an overview of a significant number of genes exhibiting distinct gene expression patterns within nine samples (three donors, three groups per donor). Of note, clustering analysis revealed that the Pre-culture and Post-culture conditions displayed different gene signatures. Interestingly, despite notable differences in phenotype (such as viability after long-term culture)^5^, Post-culture_Conventional and Post-culture_Microwell exhibited similarity in their gene expression profiles. Fig. 2b shows the total counts of 18,244 protein-coding genes across the three groups (Pre-culture, Post-culture_Conventional, and Post-culture_Microwell). The RNA-seq data revealed 16,602 genes shared among the three groups. Interestingly, the number of genes exclusively shared between Post-culture groups (ie, Post-culture_Conventional and Post-culture_Microwell, 390 genes) was higher than that shared exclusively between Pre-culture and Post-culture_Conventional (156 genes) or Pre-culture and Post-culture microwell (301 genes). This supports the finding above that Post-culture samples in different culture methods exhibited more similarity in gene signature compared to the Pre-culture samples. The protein-coding gene counts in Pre-culture versus Post-culture; the RNA-seq data showed 17,059 genes shared between the two groups (Fig. 2c). In the following analysis, we conducted subsequent detailed comparisons of gene expressions and pathway analysis between groups: (1) Pre-culture versus Post-culture_Conventional, (2) Pre-culture versus Post-culture_Microwell, and (3) Post-culture_Conventional versus Post-culture_Microwell.

### Downregulation of the metal-ion-related pathways is the key phenomenon in the long-term cultured islets regardless of the culture methods

#### Post-culture_Conventional versus Pre-cultured islets

**Figure 3.**
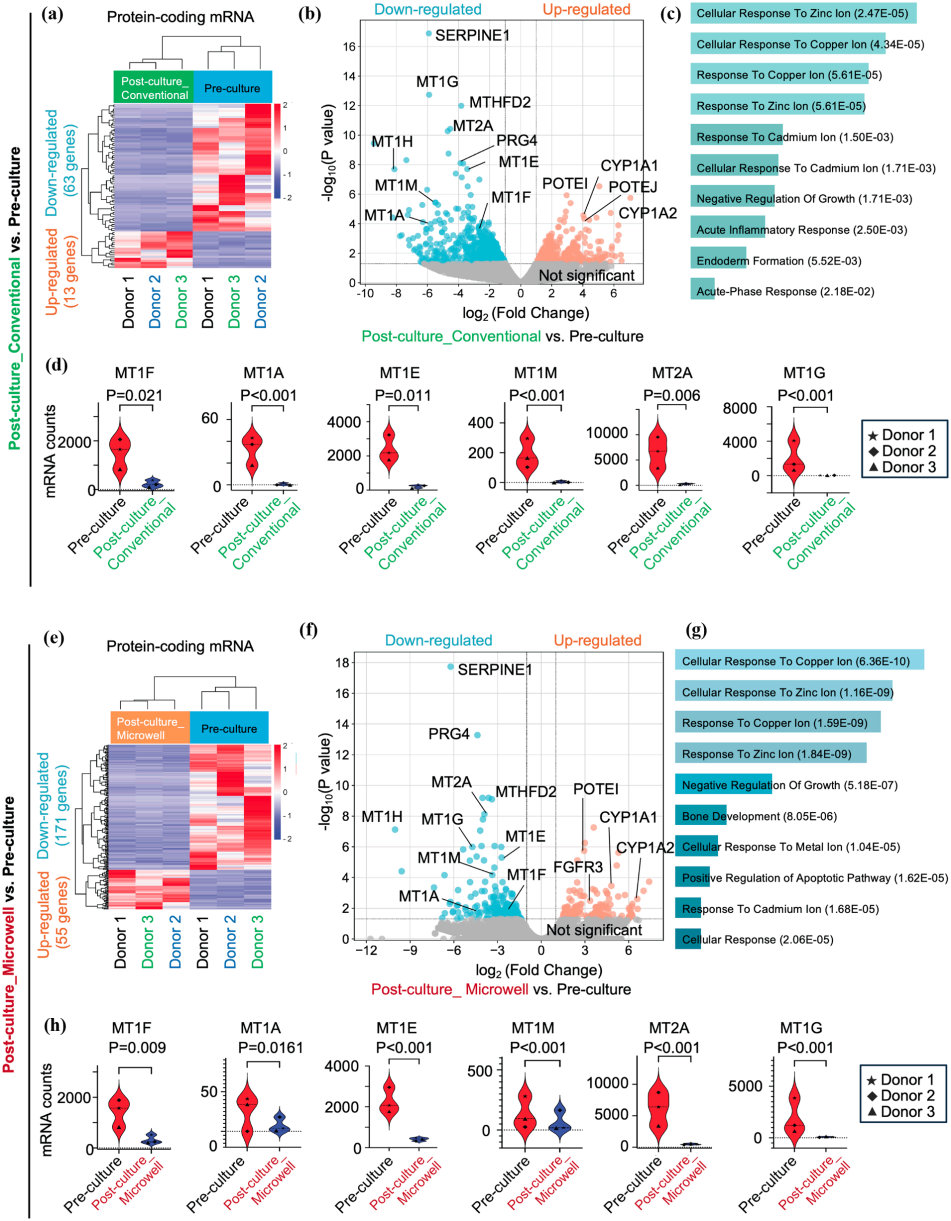
Downregulation of metal ion-related pathways in long-term cultured islets. Analyses comparing Post-culture_Conventional versus Pre-culture are presented in (a)–(d), and analyses comparing Post-culture_Microwell versus Pre-culture are presented in (e)–(h). (a & e) Heatmap displaying genes with significantly altered relative expression between the groups, with clustering analysis. Donor IDs are indicated at the bottom in distinct colors. (b & f) Volcano plot analysis illustrating differentially expressed genes in Post-culture_Microwell versus Pre-culture. Representative gene names that showed significant changes are labeled on the graph. (c & g) Enriched biological functions in GO terms, predominantly related to heavy metal ion-associated functions, based on significantly altered genes. (d & h) Violin plot analysis of metallothionein family mRNA expression across groups. Donor IDs are indicated using different markers in the figure. *p < 0.05; **p < 0.01.

We analyzed gene signatures between Post-culture_Conventional versus Pre-cultured islets. Fig. 3a shows a heatmap demonstrating the relative expression of 81 protein-coding genes that were significantly altered in these samples. We identified more downregulated genes in Post-culture_Conventional versus Pre-cultured islets, compared to upregulated genes (63 and 18 genes, respectively). The volcano plots analysis further illustrates this finding, with significantly downregulated genes including *SERPINE1*, *MT1G*, *MTHFD2* and *MT2A* (Fig. 3b). The enriched biological functions based on these genes in Gene Ontology (GO) terms are predominantly related to cellular responses to metal ions, including zinc, copper, and cadmium (Fig. 3c, Supplementary Table S3). In fact, the metallothionein (MT) family is known for regulating intracellular heavy metal homeostasis^24,25^. In our analysis, the mRNA expression levels of several MT genes—including *MT1F*, *MT1A*, *MT1E*, *MT1M*, *MT2A*, and *MT1G*—were significantly downregulated in RNA collected from Post-culture_Conventional samples compared to Pre-culture samples (Fig. 3d). The upregulated genes were enriched in multiple biological functions with Long-Chain Fatty Acid Biosynthetic Process (GO:0042759) as the most significant enriched function (Supplementary Table S3), however, its statistical significance (*p*-values) was three orders of magnitude lower compared to the most enriched function among the downregulated genes.

#### Post-culture_Microwell versus Pre-cultured islets

Next, we analyzed gene signatures between Post-culture_Microwell and Pre-cultured islets. Fig. 3e presents a heatmap illustrating the relative expression of 226 genes that were significantly altered in these samples. Similar to the analysis of Post-culture_Conventional versus Pre-culture, we identified more downregulated genes in Post-culture_Microwell compared to Pre-culture than upregulated genes (171 and 55 genes, respectively). The volcano plot analysis further supported these findings and identified significantly downregulated genes, including *SERPINE1*, *PRG4*, and several MT genes (Fig. 3f). Interestingly, these genes were also identified in the previous comparison between Post-culture_Conventional and Pre-cultured islets, which aligns well with the findings in Fig. 2, demonstrating the similarity in gene signatures between long-term cultured islets (ie, Post-culture_Conventional and Post-culture_Microwell). Gene enrichment analysis revealed that biological functions related to heavy metal ions were predominantly enriched (Fig. 3g, Supplementary Table S4). Furthermore, we analyzed the mRNA expression levels of MT family genes, demonstrating their downregulation in Post-culture_Microwell compared to Pre-cultured islets (Fig. 3h). The complete lists of differentially expressed genes are provided in Supplementary Table S5 for downregulated genes and Supplementary Table S6 for upregulated genes.

These findings highlight two key points: (1) Regardless of culture conditions (even though microwell plate culture demonstrated improved viability), long-term culture induces major changes in gene expression signatures; and (2) Heavy metal ion-related genes are predominantly downregulated in long-term cultured islets, a shared phenomenon observed in both Post-culture_Conventional and Post-culture_Microwell conditions. Toward the goal of maintaining islet characteristics during long-term culture, this could be a critical finding that cannot be identified solely based on the viability phenotype of cultured islets.

Additionally, the Post-culture_Microwell, but not the Post-culture_Conventional, showed an enriched downregulated apoptosis pathway when compared to Pre-culture (Fig. 3c, g). This may indicate a potential protective effect of the microwell over conventional culture, as demonstrated by the prevention of cellular damage in long-term cultured islets on the microwell.

**Figure 4.**
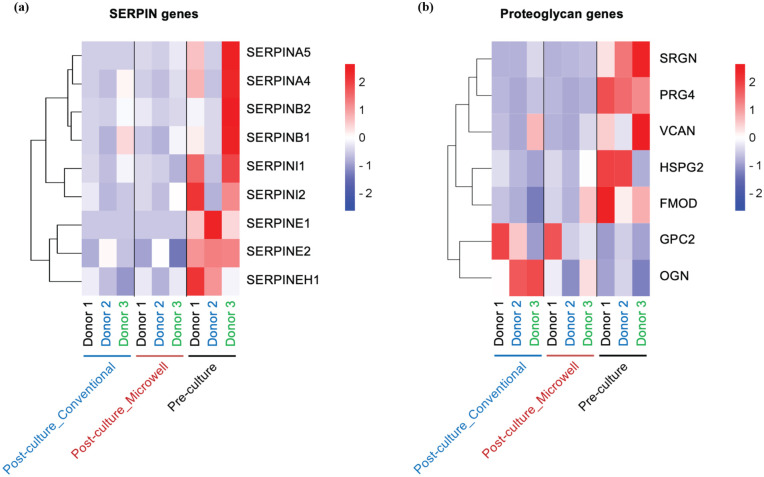
Downregulated genes other than metallothioneins in long-term cultured islets versus pre-culture. Two representative downregulated genes, aside from metallothioneins identified in our dataset, are presented in a heatmap. (a) mRNA expression of the *SERPIN* gene family, demonstrating an overall significant decrease under post-culture conditions. (b) mRNA expression of proteoglycan-related genes. Donor IDs are indicated at the bottom using distinct colors.

Other important findings include the significantly downregulated genes, such as *SERPINE1* and *PRG4*. These genes were identified in both comparisons (Fig. 3b, f). *SERPINE1* belongs to the serine protease inhibitor (SERPIN) family, which is associated with fibrinolysis, extracellular matrix remodeling, and inflammation fibrosis^26–28^. Notably, not only *SERPINE1* but also multiple subtypes of the SERPIN family are significantly downregulated in long-term cultured islets (Fig. 4a). PRG4, Proteoglycan 4, which encodes lubricin—a mucin-like proteoglycan. Proteoglycans constitute a large family with multiple classes. Among them, PRG4 was the only gene significantly altered between pre-culture and post-culture conditions in our dataset, and there was no consistent trend in gene expression changes across islet culture conditions (Fig. 4b).

#### Post-culture_Conventional versus Post-culture_Microwell

**Figure 5.**
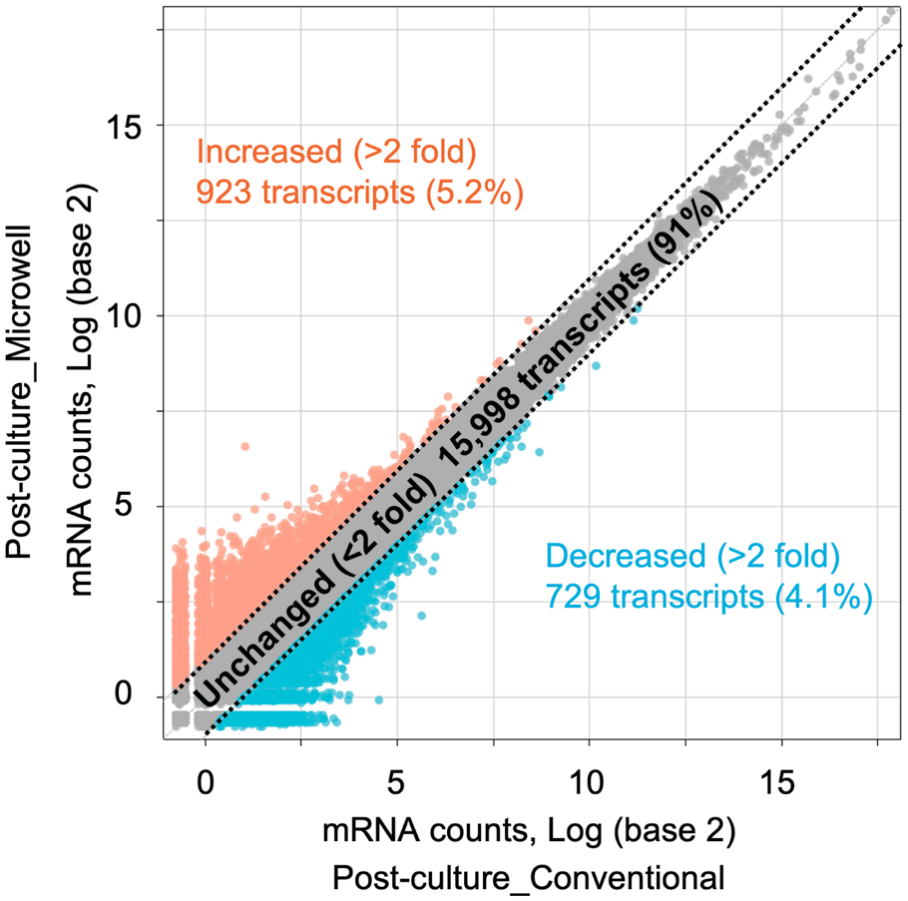
Scatter plot of differentially expressed genes between Post-culture_Conventional versus Post-culture_Microwell. Genes showing increased expression in Post-culture_Microwell compared to Post-culture_Conventional are plotted in orange, while downregulated genes are shown in blue. The dotted lines indicate transcripts with more than a 2-fold increase or decrease in mRNA expression levels.

Finally, we analyzed the gene signatures between Post-culture_Conventional and Post-culture_Microwell. The scatterplot shows the correlation in 17,650 mRNA transcripts expressed in both conditions, demonstrating the similarity of the gene signatures between the two groups, with 15,998 genes (91%) remaining unchanged based on the criterion of a >2-fold change in mRNA counts (Fig. 5). No transcript was statistically different between the two conditions after adjusting the *p*-value, and no significant biological functions were identified in the gene enrichment analysis, further supporting this finding. Specifically, when analyzed using an unpaired *t*-test, 53 transcripts were identified with a *p*-value < 0.05 (34 increased and 19 decreased in Post-culture_Microwell vs Pre-culture) (Supplementary Fig. S1).

## Discussion

This study reports genome-wide sequencing of human islets during long-term culture over two weeks, based on the newly developed culture method of isolated primary human islets on the microwells^5^, addressing a previously unmet challenge. The use of microwell culture effectively maintained islet viability by physically separating individual islets, thereby alleviating diffusion limitations caused by islet fusion during prolonged culture in conventional method. However, interestingly, we observed minimal differences of gene signature between culture methods, despite distinct outcomes in viability. Rather, long-term cultured islets, irrespective of the platform used, significantly altered their gene expression profiles compared to the pre-cultured islets. Notably, heavy metal-ion-related pathways, including zinc ion homeostasis, were downregulated in long-term cultured islets. These findings highlight intra-islet metal ion regulation as a potential therapeutic target for preserving the metabolic fitness of human islets during extended culture.

The downregulation of metal ion–related pathways was primarily attributed to the significant downregulation of the MT gene family. MTs play critical roles in metal detoxification, antioxidant defense, and the regulation of cellular redox homeostasis, largely due to their ability to chelate heavy metal elements through their thiol content^24,25^. Metal ions, such as zinc, are essential for pancreatic islet cell function; zinc is highly concentrated in islets and contributes to insulin synthesis and secretion^25,29,30^. MTs are key players in these processes, with MT1 and MT2 identified as the dominant subtypes in human islets^24^. Furthermore, MTs are known to protect β-cells from oxidative stress, thereby playing a preventative role in diabetes development^31^. The observed downregulation of these pathways in our study suggests a correlation with the loss of islet characteristics, including impaired metabolism during long-term culture. However, we acknowledge that MT expression is influenced by multiple reasons of cellular stress. Therefore, the downregulation of MT genes observed here may not solely indicate β-cell identity loss but could also reflect secondary effects of general culture stress.

Interestingly, there are contradictory reports regarding MT expression and its impact on β-cell function. For example, reduced MT1 expression has been associated with improved glucose-stimulated insulin secretion (GSIS)^32^, aligning with our previous findings that long-term cultured human islets improved GSIS after two weeks^5^. This highlights the complexity of MT-related pathways and their dual roles in regulating β-cell function, viability and metabolism under varying conditions. To prove the dual role of MTs, broader functional phenotypes beyond those already reported will need to be examined in association with MT modulation in long-term cultured islets.

Some MT genes, such as *MT2A* and *MT1E*, are expressed at higher levels in adult islets compared to fetal islets^24,33^, suggesting their involvement in the differentiation and maturation of islets. By analyzing previously deposited RNA-seq datasets during the differentiation of stem cell-derived beta cells^34,35^, we observed an upregulation of metal ion pathways during the differentiation process. The downregulation of metal ion pathways observed in our dataset may indicate the loss of mature β-cell properties under inappropriate culture conditions.

Comprehensive analysis identified metal ion pathways as a potential target for improving islet culture and preserving metabolic function. To counteract their downregulation, possible interventions include supplementing media with metal ions, using chelated forms to enhance uptake, or incorporating binding proteins such as albumin or MTs. Genetic tools such as CRISPR could also be employed to upregulate key metal-ion transport genes and optimize long-term culture conditions. Since metal ion homeostasis pathways are highly conserved across species, these strategies may also be applicable to human stem cell–derived and xenogeneic islets. Experimental evidence supports the benefits of such interventions. For example, zinc supplementation in beta cells increased the expression of MT genes, enhancing β-cell function and survival^36^. Similarly, in isolated rat islets, supplementation with ZnCl_2_ elevated *Mt1A* and *Mt2A* expression while significantly reducing β-cell apoptosis^37^. Furthermore, maintaining MT gene expression in islets has been linked to a narrow optimal glucose concentration in the culture media^32^, which highlights the importance of carefully selecting glucose levels. Critically, no studies to date have demonstrated the effects of zinc or MT interventions on long-term cultured islets. Therefore, optimizing these approaches represents an important next step toward improving transplantation outcomes using long-term cultured islets. Additional MT stimulants include stress-related factors such as reactive oxygen species generation^38^, proinflammatory cytokines^39^, hypoxic stress^40^, and glucocorticoid hormones^41^. These findings suggest that maintaining islets in stable conditions lacking stimuli may inadvertently decrease MT expression. Applying intermittent stimuli during the culture period could help sustain intra-islet MT levels; however, excessive stress can cause islet damage, emphasizing the need for a balanced approach to treatment.

Besides the alterations in *MT* gene family expression, which contributed to the enrichment of metal ion–related pathways in our study, we also identified several significantly downregulated genes of interest, although they did not contribute to the enrichment of any specific biological function.

*SERPINE1*, also known as plasminogen activator inhibitor-1 (PAI-1), was consistently downregulated in long-term cultured islets under both Post-culture_Conventional and Post-culture_Microwell conditions. SERPIN has been identified as having multifunctional roles, including regulation of fibrinolysis, extracellular matrix remodeling, inflammation, cell migration, cellular senescence, and tissue fibrosis^26–28^. In the context of islets and β-cells, *SERPINE1* has been implicated in β-cell damage and may impair insulin sensitivity and islet microvasculature^42–44^. Interestingly, several subtypes of the SERPIN family were also downregulated in long-term cultured islets. Therefore, targeting a broad spectrum of the SERPIN family may be an effective strategy for preserving islet characteristics during long-term culture. Potential interventions include the use of TGF-β1, which has been shown to act as an upstream signaling modulator of *SERPINE1* expression in multiple cell types^45,46^.

PRG4 was also consistently downregulated in long-term cultured islets. PRG4, a member of the proteoglycan family, encodes lubricin—a mucin-like proteoglycan originally identified in articular cartilage—and has recently been implicated in the regulation of inflammatory responses^47,48^. While other types of proteoglycans, such as heparan sulfate proteoglycans are known to be lost during the isolation of islets from the pancreas and have been associated with impaired islet function^49,50^, few studies have examined the role of PRG4 in islet function or survival. The consistent downregulation of PRG4 in long-term cultured islets suggests that it could serve as a novel marker for losing islet characteristics or the onset of cellular senescence. Although both SERPINE1 and PRG4 were consistently downregulated in long-term cultured islets, the functional significance of these changes remains uncertain. Whether their reduced expression reflects an adaptive response to culture stress, a pathological decline in critical support functions, or incidental shifts without direct relevance to islet biology remains unclear. Functional validation of these genes, including protein expression and localization as well as correlation with islet functional assays, will be required to clarify their role. In addition, studies with larger donor will help to establish correlations with relevant pathways and to better define their functional significance.

There was a significant difference in viability between the two post-culture conditions (Post-culture_Conventional vs Post-culture_Microwell); however, the gene expression profiles were predominantly similar. Potential considerations for the divergence between viability outcomes and transcriptomic profiles include post-transcriptional regulation that decouples mRNA levels from protein abundance (eg, zinc transporters or MTs), metabolic flux differences that alter physiological function without large-scale transcriptional shifts, and bulk RNA-seq may obscure subtle changes in specific islet cell composition. In addition, reduced stress in microwells may enhance protein stability and cell survival despite similar transcriptional trends. Despite this, a few interesting genes emerged from the comparison. For instance, *PRSS33* was upregulated in the Post-culture_Microwell group compared to Post-culture_Conventional. *PRSS33* is involved in protein kinase C signaling and proteolysis^51–53^. Few studies have demonstrated a direct association between this gene and pancreatic islets. Although the mechanisms underlying the differential expression of the gene between the two post-culture conditions remain unclear, they may serve as potential markers of islet survival or damage. Additional validation studies are required to confirm their roles.

Limitations of this study include the small donor sample size (n = 3, all female), which reduces statistical power and introduces the possibility of sex-related bias. The use of bulk RNA-seq prevents cell population-specific analyses, such as beta-cell-specific gene expression changes. Since this study relies on genome sequencing, integrating multiple omics approaches could help elucidate the detailed mechanisms underlying islet characteristic changes and identify strategies to maintain cellular function during long-term culture. Additionally, our analysis focused on protein-coding genes, but non-coding RNAs are also critical regulators of gene expression and cellular function. miRNAs, for example, modulate β-cell differentiation, insulin secretion, and stress responses^54–56^. Further analysis of non-coding RNAs could uncover novel biomarkers and therapeutic targets to maintain islet characteristics during the culture.

Future experimental methods could include proteomics to analyze the protein profiles of cultured islets and the secretome of culture media, which will provide insights into the functional state of the islets. Sampling at multiple timepoints during the two-week culture period could reveal dynamic changes in gene expression and protein activity over time. Such time-course analyses could also help identify critical windows for potential interventions to preserve islet function and viability. Finally, while our microwell platform was designed to accommodate higher seeding densities relevant to current clinical applications, we acknowledge that this may contribute to microdamage and downregulation of metal ion pathways. Adjusting culture conditions by lowering density and/or increasing the frequency of medium exchange—particularly with pharmacological supplementation of metal ions—could help to better preserve these pathways.

## Supplemental Material

sj-jpg-1-cll-10.1177_09636897251390960 – Supplemental material for Long-term culture of human pancreatic islets reveals reduced metal ion pathways in their gene signature Supplemental material, sj-jpg-1-cll-10.1177_09636897251390960 for Long-term culture of human pancreatic islets reveals reduced metal ion pathways in their gene signature by Hiroyuki Kato, Tara K. Sigdel, Mona Sheta, Keiko Omori, Meirigeng Qi, Fouad Kandeel and Hirotake Komatsu in Cell Transplantation

sj-xls-2-cll-10.1177_09636897251390960 – Supplemental material for Long-term culture of human pancreatic islets reveals reduced metal ion pathways in their gene signature Supplemental material, sj-xls-2-cll-10.1177_09636897251390960 for Long-term culture of human pancreatic islets reveals reduced metal ion pathways in their gene signature by Hiroyuki Kato, Tara K. Sigdel, Mona Sheta, Keiko Omori, Meirigeng Qi, Fouad Kandeel and Hirotake Komatsu in Cell Transplantation

sj-xls-3-cll-10.1177_09636897251390960 – Supplemental material for Long-term culture of human pancreatic islets reveals reduced metal ion pathways in their gene signature Supplemental material, sj-xls-3-cll-10.1177_09636897251390960 for Long-term culture of human pancreatic islets reveals reduced metal ion pathways in their gene signature by Hiroyuki Kato, Tara K. Sigdel, Mona Sheta, Keiko Omori, Meirigeng Qi, Fouad Kandeel and Hirotake Komatsu in Cell Transplantation

sj-xls-4-cll-10.1177_09636897251390960 – Supplemental material for Long-term culture of human pancreatic islets reveals reduced metal ion pathways in their gene signature Supplemental material, sj-xls-4-cll-10.1177_09636897251390960 for Long-term culture of human pancreatic islets reveals reduced metal ion pathways in their gene signature by Hiroyuki Kato, Tara K. Sigdel, Mona Sheta, Keiko Omori, Meirigeng Qi, Fouad Kandeel and Hirotake Komatsu in Cell Transplantation

sj-xls-5-cll-10.1177_09636897251390960 – Supplemental material for Long-term culture of human pancreatic islets reveals reduced metal ion pathways in their gene signature Supplemental material, sj-xls-5-cll-10.1177_09636897251390960 for Long-term culture of human pancreatic islets reveals reduced metal ion pathways in their gene signature by Hiroyuki Kato, Tara K. Sigdel, Mona Sheta, Keiko Omori, Meirigeng Qi, Fouad Kandeel and Hirotake Komatsu in Cell Transplantation

sj-xls-6-cll-10.1177_09636897251390960 – Supplemental material for Long-term culture of human pancreatic islets reveals reduced metal ion pathways in their gene signature Supplemental material, sj-xls-6-cll-10.1177_09636897251390960 for Long-term culture of human pancreatic islets reveals reduced metal ion pathways in their gene signature by Hiroyuki Kato, Tara K. Sigdel, Mona Sheta, Keiko Omori, Meirigeng Qi, Fouad Kandeel and Hirotake Komatsu in Cell Transplantation

sj-xls-7-cll-10.1177_09636897251390960 – Supplemental material for Long-term culture of human pancreatic islets reveals reduced metal ion pathways in their gene signature Supplemental material, sj-xls-7-cll-10.1177_09636897251390960 for Long-term culture of human pancreatic islets reveals reduced metal ion pathways in their gene signature by Hiroyuki Kato, Tara K. Sigdel, Mona Sheta, Keiko Omori, Meirigeng Qi, Fouad Kandeel and Hirotake Komatsu in Cell Transplantation
