# Supplementary figures and images for: Long-term culture of human pancreatic islets reveals reduced metal ion pathways in their gene signature
Source: Cell Transplant. 2025 Nov 22;34:09636897251390960. doi: 10.1177/09636897251390960 (PMC12640451; doi:10.1177/09636897251390960)

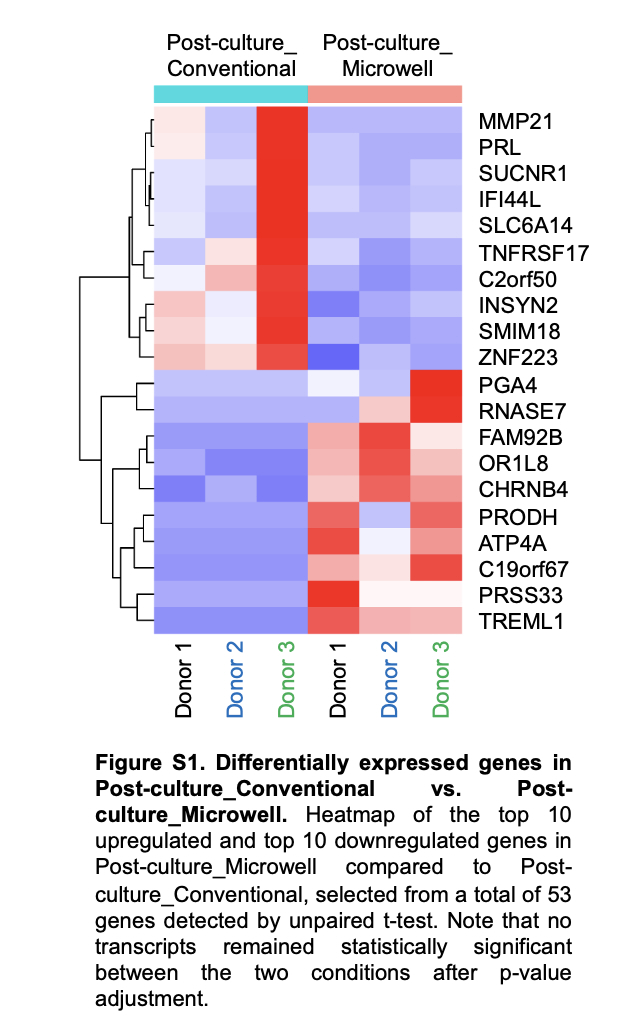

Supplement: sj-jpg-1-cll-10.1177_09636897251390960 – Supplemental material for Long-term culture of human pancreatic islets reveals reduced metal ion pathways in their gene signature [file sj-jpg-1-cll-10.1177_09636897251390960.jpg]
